# Integrative Analysis of Biomarkers and Mechanisms in Adamantinomatous Craniopharyngioma

**DOI:** 10.3389/fgene.2022.830793

**Published:** 2022-03-30

**Authors:** Da Lin, Wenyue Zhao, Jun Yang, Hao Wang, Hongbing Zhang

**Affiliations:** ^1^ Department of Neurosurgery, Beijing Luhe Hospital, Capital Medical University, Beijing, China; ^2^ Institute of Radiation Medicine, Chinese Academy of Medical Sciences and Peking Union Medical College, Tianjin, China

**Keywords:** adamantinomatous craniopharyngioma (ACP), differentially expressed immune-related genes, hub gene, diagnostic markers, therapeutic targets

## Abstract

Craniopharyngioma is a benign tumor, and the predominant treatment methods are surgical resection and radiotherapy. However, both treatments may lead to complex complications, seriously affecting patients’ survival rate and quality of life. Adamantinomatous craniopharyngioma (ACP), as one of the histological subtypes of craniopharyngioma, is associated with a high incidence and poor prognosis, and there is a gap in the targeted therapy of immune-related genes for ACP. In this study, two gene expression profiles of ACP, namely GSE68015 and GSE94349, were downloaded from the Gene Expression Omnibus (GEO) database. The differentially expressed genes (DEGs) were identified by the Limma package, and 271 differentially expressed immune-related genes (DEIRGs) were obtained from the Immport database. The gene ontology (GO), Kyoto Encyclopedia of Genes and Genomes (KEGG), and Gene Set Enrichment Analysis (GSEA) were performed for annotation, visualization, and integrated discovery. Five hub genes, including CXCL6, CXCL10, CXCL11, CXCL13, and SAA1, were screened out through protein-protein interaction (PPI) network interaction construction. Two diagnostic markers, namely S100A2 and SDC1 (both of which have the Area Under Curve value of 1), were screened by the machine learning algorithm. CIBERSORT analysis showed that M2 macrophages, activated NK cells, and gamma delta T cells had higher abundance in ACP infiltration, while CD8+ T cells, regulatory T cells, and Neutrophils had less abundance in ACP infiltration. The expression of gamma delta T cells was positively correlated with CXCL6, S100A2, SDC1, and SAA1, while CD8+ T cells expression was negatively correlated with CXCL6, S100A2, SDC1, and CXCL10. ACP with high CXCL6 showed remarkable drug sensitivity to Pentostatin and Wortmannin via CellMiner database analysis. Our results deepened the understanding of the molecular immune mechanism in ACP and provided potential biomarkers for the precisely targeted therapy for ACP.

## Introduction

Craniopharyngioma (CP) is a rare epithelial neoplasm derived from the remnants of Rathke’s pouch (Prabhu and Brown, 2005). Due to its proximity to critical neurovascular structures such as the optic nerve, pituitary, hypothalamus, and internal carotid artery, pre-and postoperative complications such as visual impairment, endocrine deficiencies, and hypothalamic dysfunctions frequently occur after CP, thus leading to a low survival rate and poor quality of life in CP patients (Müller et al., 2019).

Currently, the treatment of CP mainly includes gross total resection (GTR) and subtotal resection (STR) followed by adjuvant radiotherapy (RT), but it is controversial which of these two methods is more advantageous. Although GTR reduces the possibility of recurrence to the greatest extent, it is associated with a higher risk of neurological complications such as hypothalamus injury. By contrast, STR + RT may retain patients’ neurological function but is associated with a higher risk of recurrence and radiotherapy complications (Aggarwal et al., 2013; Shi X.e. et al., 2017). Therefore, a novel and rational treatment safer than the current treatment methods is needed for this challenging tumor.

The histological subtypes of CP include papillary craniopharyngioma (PCP) and adamantinomatous craniopharyngioma (ACP), and the incidence of ACP is higher than that of PCP. More than 90% of PCP carries BRAF mutations and BRAF V600E protein expression (Malgulwar et al., 2017). Molecular-targeted therapy with BRAF and MEK inhibitors for PCP with BRAF mutation may achieve the goal of reducing tumor volume, thus becoming a neoteric treatment for PCP (Brastianos et al., 2014, 2016; Roque and Odia, 2017; Juratli et al., 2019). By contrast, studies have reported that activation of the Wnt pathway by CTNNB1 mutations and positive β-catenin protein expression is the pathogenic mechanism of ACP, but there is still no clear molecular therapeutic target for ACP at present.

Immune-related genes play crucial roles in the tumor immune microenvironment (Li et al., 2020), and immunotherapy is a validated and critically important approach for treating patients with cancer (Hegde and Chen, 2020). Coy et al. found that PD-1/PD-L1 played an important role in the pathogenesis of ACP and suggested that PD-1/PD-L1 could be a potential immunotherapy target (Coy et al., 2018); however, the drug sensitivity of this target was not tested in their study. There are few studies on ACP immune-related differential genes, so it is necessary to study further the expression and mechanism of ACP immune-related differential genes to find feasible immunotherapy targets.

In this study, we downloaded two ACP gene expression profile datasets from Gene Expression Omnibus (GEO) database and screened out the differentially expressed immune-related genes (DEIRGs). We analyzed the biological functions of DEIRGs by enrichment analysis and used protein-protein interaction (PPI) network interaction and machine learning algorithm to screen hub genes and diagnostic markers. We calculated the abundance of immune infiltrating cells in tumor cells and analyzed the correlation between the immune infiltrating cells and the biomarkers. In addition, we used the CellMiner database to predict the drug sensitivity of the screened biomarkers. Our findings on the therapeutic targets based on the molecular immune mechanism could be further employed as promising biomarkers or treatment targets for ACP.

## Materials and Methods

### Data Collection and Preprocessing

ACP expression profiles datasets GSE68015 (Gump et al., 2015) and GSE94349 were downloaded from the GEO database (https://www.ncbi.nlm.nih.gov/geo/) (Barrett et al., 2013). The species selected were Homo sapiens, and the platform was GPL570 [HG-U133_Plus_2] Affymetrix Human Genome U133 Plus 2.0 Array. The GSE68015 dataset included 15 ACP samples and 16 control brain tissues, and the GSE94349 dataset included 9 ACP samples and 17 control brain tissues. The original data of GSE68015 and GSE94349 datasets were read through the DealGPL570 package, background correction and data normalization were carried out, and the gene expression matrices of the two datasets were obtained, respectively.

### Identification of DEIRGs

Differentially expressed genes (DEGs) in the GSE68015 and GSE94349 datasets were screened by the “Limma” package (Ritchie et al., 2015), and volcano maps of DEGs were performed to explore the differentially expressed DEGs using the “ggplot2” package. DEGs satisfy *p*-value < 0.05 and |log2FC| >1(The cut-off criteria for statistical significance were a log fold change (FC) of greater than one and a *p* value of less than 0.05). The immune-related genes (IRGs) list was downloaded from the ImmPort database (https://immport.niaid.nih.gov) (Bhattacharya et al., 2018), and then the DEIRGs were extracted from DEGs.

### Functional and Pathway Enrichment Analysis

We used the “clusterProfiler” package (Yu et al., 2012) to perform Gene Ontology (GO) (Ashburner et al., 2000) and Kyoto Encyclopedia of Genes and Genomes (KEGG) (Kanehisa et al., 2017) enrichment analysis on DEIRGs. GO analysis included annotation of biological processes (BPs), Molecular functions (MFs), and Cellular Components (CCs). Also, Gene Set Enrichment Analysis (GSEA) was performed on the gene expression matrix through the “clusterProfiler” package (Hänzelmann et al., 2013), and “c2. cp.kegg.v7.0. symbols.gmt” was selected as a reference gene set. A false discovery rate (FDR) < 0.25 and *p* < 0.05 was considered to be significantly enriched.

### PPI Network Construction and Identification of Hub Genes

These DEIRGs were put into the STRING database (https://www.string-db.org/) (von Mering et al., 2003) to obtain the interaction relationships, and Cytoscape software (Shannon et al., 2003) was used to visualize the interaction relationships of DEIRGs with interaction scores greater than or equal to 0.9. The hub genes in the network were mined based on Molecular Complex Detection (Bandettini et al., 2012) and cytoHubba (Chin et al., 2014).

### Screening and Validation of Diagnostic Markers

We used two machine learning algorithms, namely Random Forest and least absolute shrinkage and selection operator (LASSO) regression, to screen diagnostic markers in the GSE68015 dataset. The “glmnet” package (Engebretsen and Bohlin, 2019) was used to implement the LASSO algorithm, and the “randomForest” package (Alderden et al., 2018) was used to implement the Random Forest algorithm. Finally, the intersection of diagnostic markers obtained by the two algorithms was taken as the final result, and receiver operating characteristic (ROC) analysis was performed for each diagnostic marker in the GSE94349 dataset to verify its accuracy.

### Relationship Between Immune Cell Infiltration and Diagnostic Markers

We uploaded the gene expression matrix data to CIBERSORT to obtain the immune cell infiltration matrix. A heatmap was made using the R language “pheatmap” package (https://CRAN.R-project.org/package=pheatmap) to show the distribution of 22 kinds of immune infiltrating cells in each sample. The “ggplot2” package was used to map the distribution of immune cells in the samples. The “corrplot” package draws a correlation heatmap to visualize the correlation of 22 immune infiltrating cells. The “ggplot2” package was used to create a violin diagram to visualize differences in the infiltration of 22 immune cells. We analyzed the correlation between the selected hub genes, the diagnostic markers, and the immune infiltrating cells. The results were visualized using the “ggplot2” package (Ito and Murphy, 2013).

### Drug Sensitivity Analysis of Hub Gene and Diagnostic Markers

Data of genes and drug sensitivity were downloaded from the CellMiner database (https://discover.nci.nih.gov/cellminer/) (Shankavaram et al., 2009), and the top 16 drugs correlated with the hub genes and the diagnostic markers were selected and visualized by “corrplot” package.

## Results

### Data Preprocessing and DEIRGs Screening

**FIGURE 1:**
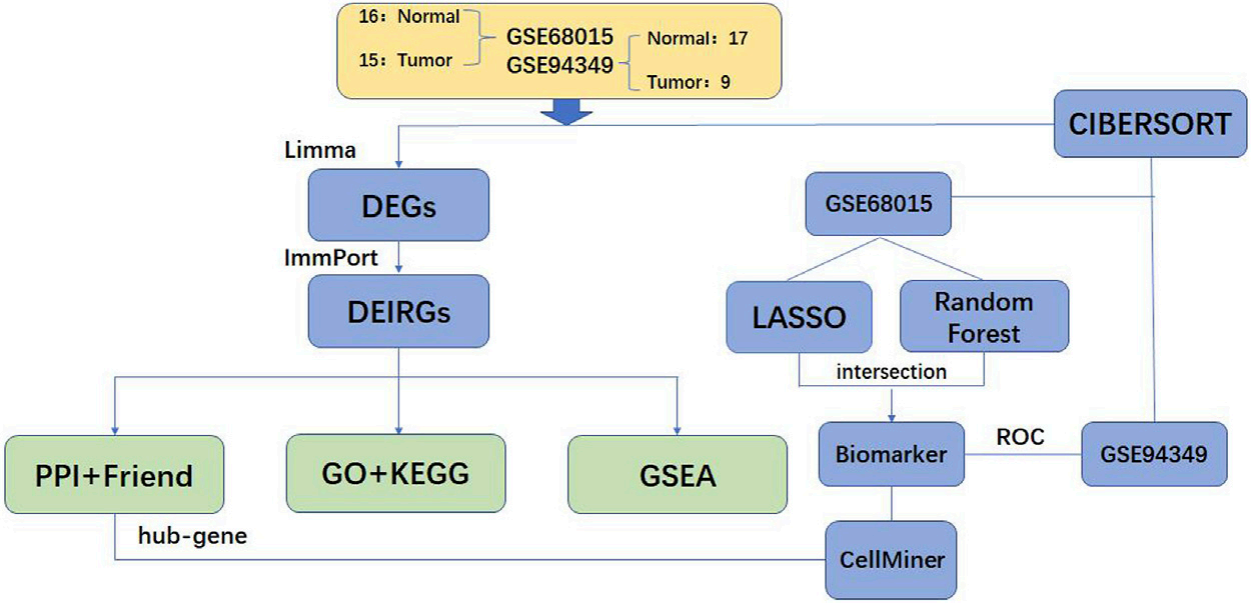
Flow chart of methodologies applied in this study.

**FIGURE 2:**
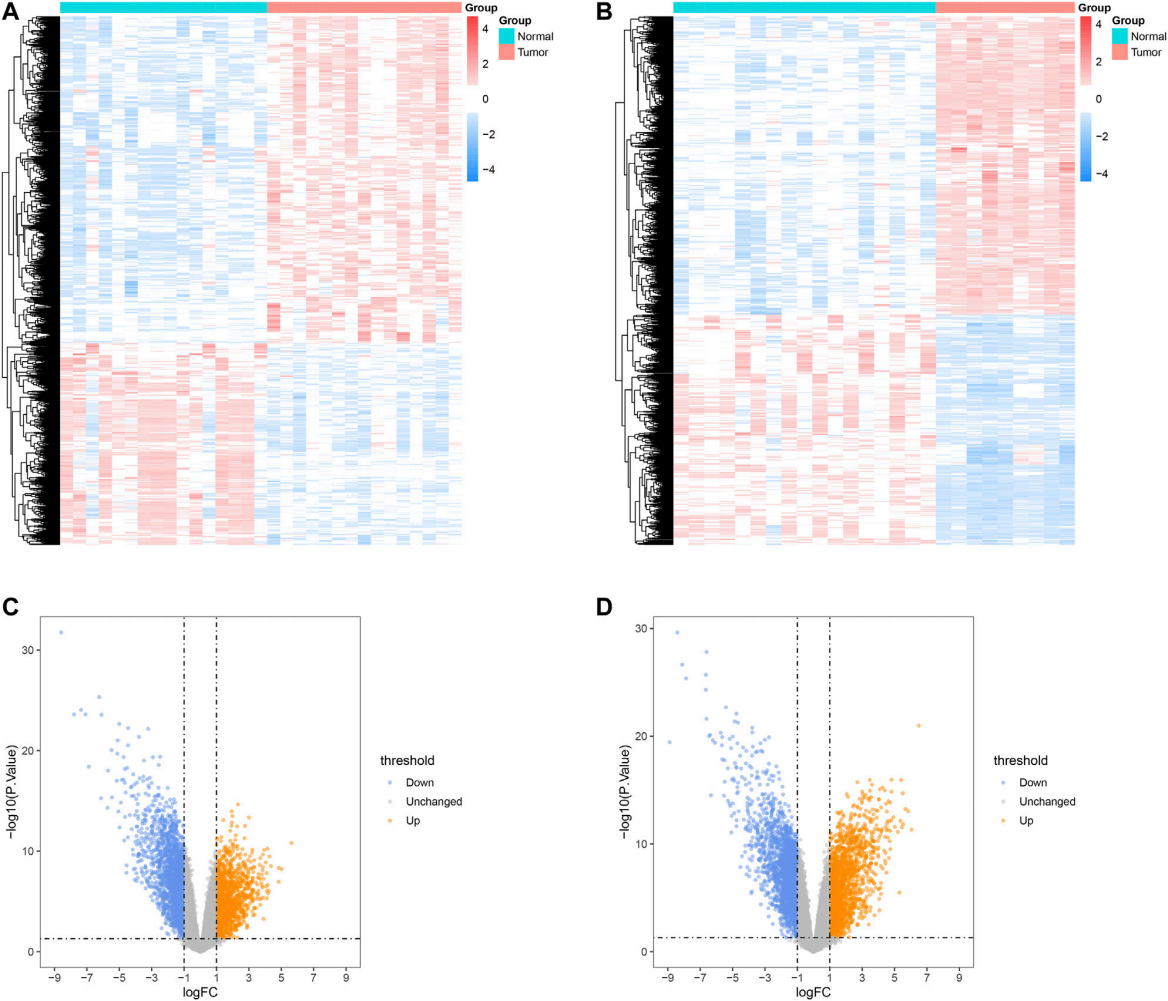
Analysis results of DEGs in ACP. Heatmaps of DEGs identified in GSE68015 **(A)** and GSE94349 **(B)**. Volcano plot analysis identifies DEGs in GSE68015 **(C)** and GSE94349 **(D)**.

The overview of the workflow is shown in Figure 1. We extracted 3,727 DEGs from the GSE68015, including 1,421 up-regulated genes and 2,306 down-regulated genes, and 4,242 DEGs from the GSE94349, including 1848 up-regulated genes and 2,394 down-regulated genes. The results of the DEGs analysis are shown in Figure 2. The intersection of DEGs and immune-related genes in these two datasets was taken to obtain 271 DEIRGs.

### Functional Correlation Analysis

**FIGURE 3:**
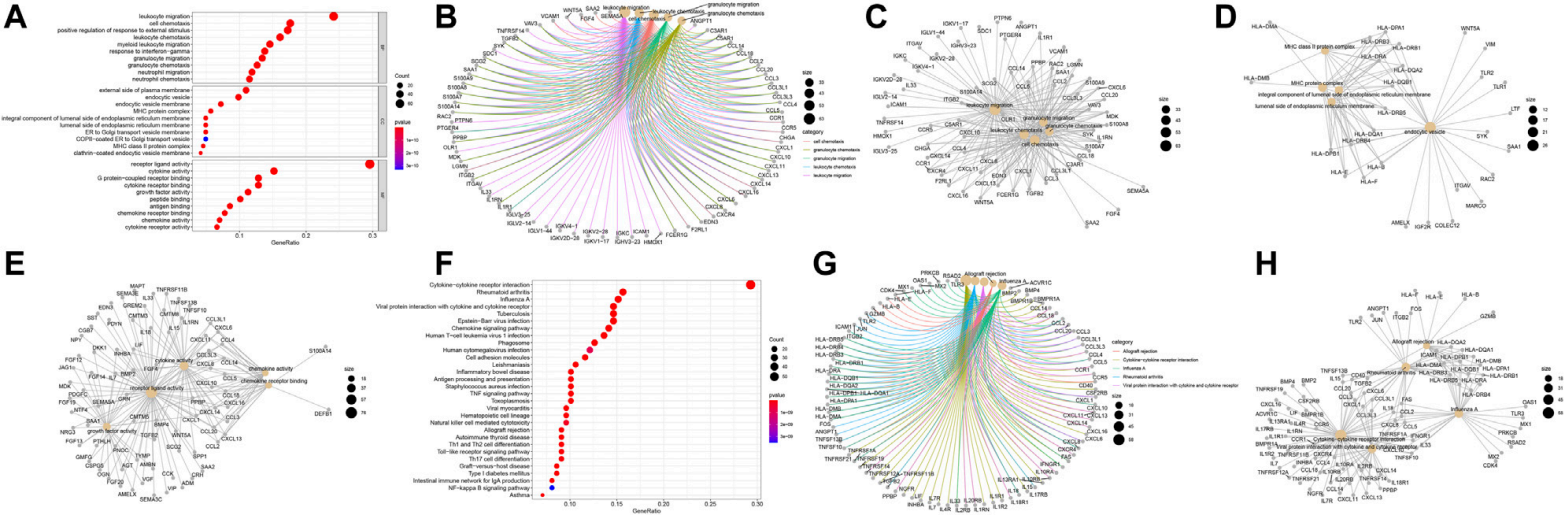
Functional correlation analysis of DEIRGs. Enrichment analysis of GO biological functions **(A)**. The X horizontal axis represents the proportion of DEGs enriched in the GO term. The bluer the color, the greater the corrected *p* value. The size of the dots represents the number of enriched genes. Other manifestations of GO enrichment analysis **(B–E)**. Enrichment analysis of KEGG pathway **(F–H)**.

**FIGURE 4:**
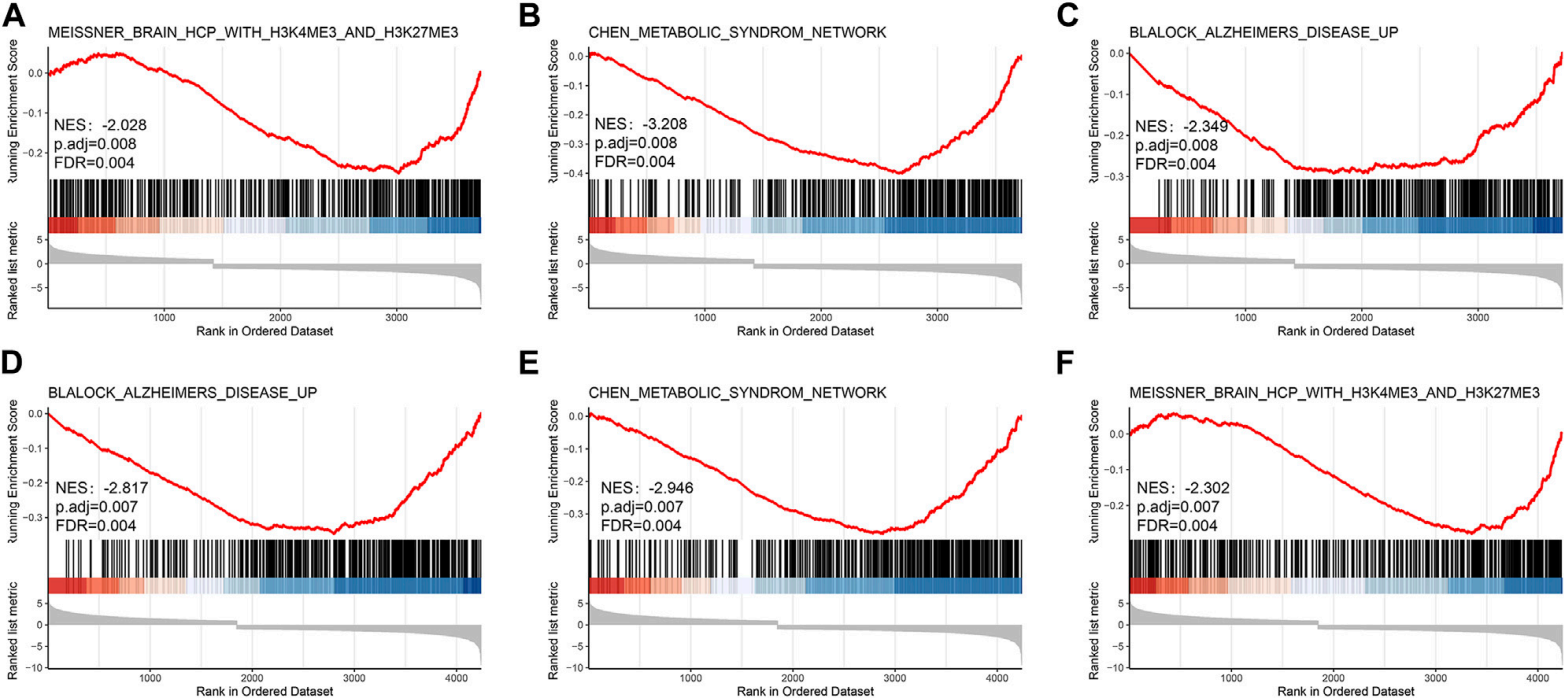
The results of the GSEA analysis in GSE68015 **(A–C)** and GSE94349 **(D–F)**.

The significance of each gene was evaluated by biological process (BP), molecular function (MF), and cellular component (CC) semantics based on GO analysis. GO analysis showed that DEIRGs were mainly associated with “leukocyte migration” and “cell chemotaxis” (Figure 3A). KEGG analysis results are shown in Figure 3F. The pathways enriched by DEIRGs mainly include “cytokine receptor interaction” and “TNF signaling pathway.” Detailed information of GO and KEGG enrichment analysis is shown in Table.1. GSEA enrichment pathways are mainly involved in the Blalock Alzheimer’s Disease Up and Chen Metabolic Syndrom Network pathways (Figure 4), and GSEA details are shown in Table.2.

**TABLE 1:** GO and KEGG pathway enrichment analysis of DEIRGs in the most significant module.

Category	Pathway ID	Pathway Description	Count in gene set	*p* Value
GO-BP	GO:0050900	Leukocyte migration	63	3.61E-42
GO-BP	GO:0030595	Leukocyte chemotaxis	42	3.01E-35
GO-BP	GO:0060326	Cell chemotaxis	46	3.29E-34
GO-BP	GO:0097530	Granulocyte migration	35	5.33E-34
GO-BP	GO:0071621	Granulocyte chemotaxis	33	2.52E-33
GO-BP	GO:0032103	Positive regulation of response to external stimulus	45	7.47E-32
GO-BP	GO:0030593	Neutrophil chemotaxis	30	2.01E-31
GO-BP	GO:0097529	Myeloid leukocyte migration	38	2.58E-31
GO-BP	GO:1990266	Neutrophil migration	31	4.92E-31
GO-BP	GO:0034341	Response to interferon-gamma	36	1.09E-29
GO-MF	GO:0048018	Receptor ligand activity	76	1.31E-57
GO-MF	GO:0005125	Cytokine activity	39	3.55E-31
GO-MF	GO:0008083	Growth factor activity	29	2.09E-23
GO-MF	GO:0042379	Chemokine receptor binding	20	1.85E-21
GO-MF	GO:0008009	Chemokine activity	18	3.51E-21
GO-MF	GO:0001664	G protein-coupled receptor binding	33	1.14E-20
GO-MF	GO:0005126	Cytokine receptor binding	33	2.22E-20
GO-MF	GO:0003823	Antigen binding	22	1.52E-15
GO-MF	GO:0004896	Cytokine receptor activity	17	3.75E-14
GO-MF	GO:0042277	Peptide binding	26	1.92E-13
GO-CC	GO:0042611	MHC protein complex	15	1.55E-22
GO-CC	GO:0042613	MHC class II protein complex	12	4.49E-20
GO-CC	GO:0071556	Endoplasmic reticulum membrane	13	1.85E-17
GO-CC	GO:0098553	lumenal side of endoplasmic reticulum membrane	13	1.85E-17
GO-CC	GO:0030139	Endocytic vesicle	26	5.86E-14
GO-CC	GO:0009897	External side of plasma membrane	29	9.47E-14
GO-CC	GO:0030666	Endocytic vesicle membrane	19	1.11E-12
GO-CC	GO:0012507	ER to Golgi transport vesicle membrane	13	1.53E-12
GO-CC	GO:0030669	Clathrin-coated endocytic vesicle membrane	11	1.04E-11
GO-CC	GO:0030134	COPII-coated ER to Golgi transport vesicle	13	3.31E-10
KEGG	hsa04060	Cytokine-cytokine receptor interaction	58	1.04E-37
KEGG	hsa05323	Rheumatoid arthritis	31	1.19E-27
KEGG	hsa04061	Viral protein interaction with cytokine and cytokine	29	6.25E-24
KEGG	hsa05330	Allograft rejection	18	9.67E-20
KEGG	hsa05164	Influenza A	30	7.37E-18
KEGG	hsa05321	Inflammatory bowel disease	20	2.37E-17
KEGG	hsa05332	Graft-versus-host disease	17	2.94E-17
KEGG	hsa04940	Type I diabetes mellitus	17	4.76E-17
KEGG	hsa05140	Leishmaniasis	21	5.85E-17
KEGG	hsa05416	Viral myocarditis	19	8.39E-17

GO, gene ontology; KEGG, kyoto encyclopedia of genes and genomes; DEIRGs, differentially expressed immune-related genes; BP, biological process; MF, molecular function; CC, cellular component.

**TABLE 2:** The results of GSEA function enrichment analysis.

GSE	Description	NES	*P*. Adjust	*Q* Values
GSE68015	MEISSNER_BRAIN_HCP_WITH_H3K4ME3_AND_H3K27ME3	−2.05116	0.008722	0.004345
GSE68015	CHEN_METABOLIC_SYNDROM_NETWORK	−3.2324	0.008722	0.004345
GSE68015	BLALOCK_ALZHEIMERS_DISEASE_UP	−2.36677	0.008722	0.004345
GSE68015	DODD_NASOPHARYNGEAL_CARCINOMA_DN	−2.45808	0.008722	0.004345
GSE68015	NABA_MATRISOME	−3.96781	0.008722	0.004345
GSE68015	DODD_NASOPHARYNGEAL_CARCINOMA_UP	−1.68793	0.008722	0.004345
GSE68015	REACTOME_DEVELOPMENTAL_BIOLOGY	−2.57766	0.008722	0.004345
GSE68015	NUYTTEN_EZH2_TARGETS_UP	−2.47255	0.008722	0.004345
GSE68015	SMID_BREAST_CANCER_BASAL_UP	−3.28168	0.008722	0.004345
GSE68015	REACTOME_INNATE_IMMUNE_SYSTEM	−2.37392	0.008722	0.004345
GSE94349	BLALOCK_ALZHEIMERS_DISEASE_UP	−2.36949	0.007431	0.003751
GSE94349	CHEN_METABOLIC_SYNDROM_NETWORK	−3.23008	0.007431	0.003751
GSE94349	MEISSNER_BRAIN_HCP_WITH_H3K4ME3_AND_H3K27ME3	−2.04056	0.007431	0.003751
GSE94349	DODD_NASOPHARYNGEAL_CARCINOMA_DN	−2.45174	0.007431	0.003751
GSE94349	NABA_MATRISOME	−3.94637	0.007431	0.003751
GSE94349	NUYTTEN_EZH2_TARGETS_UP	−2.47255	0.007431	0.003751
GSE94349	GRAESSMANN_APOPTOSIS_BY_DOXORUBICIN_DN	−1.74743	0.007431	0.003751
GSE94349	REACTOME_DEVELOPMENTAL_BIOLOGY	−2.56922	0.007431	0.003751
GSE94349	REACTOME_INNATE_IMMUNE_SYSTEM	−2.36983	0.007431	0.003751
GSE94349	BRUINS_UVC_RESPONSE_LATE	−2.23482	0.007431	0.003751

### PPI Network Construction and Hub Gene Selection

**FIGURE 5:**
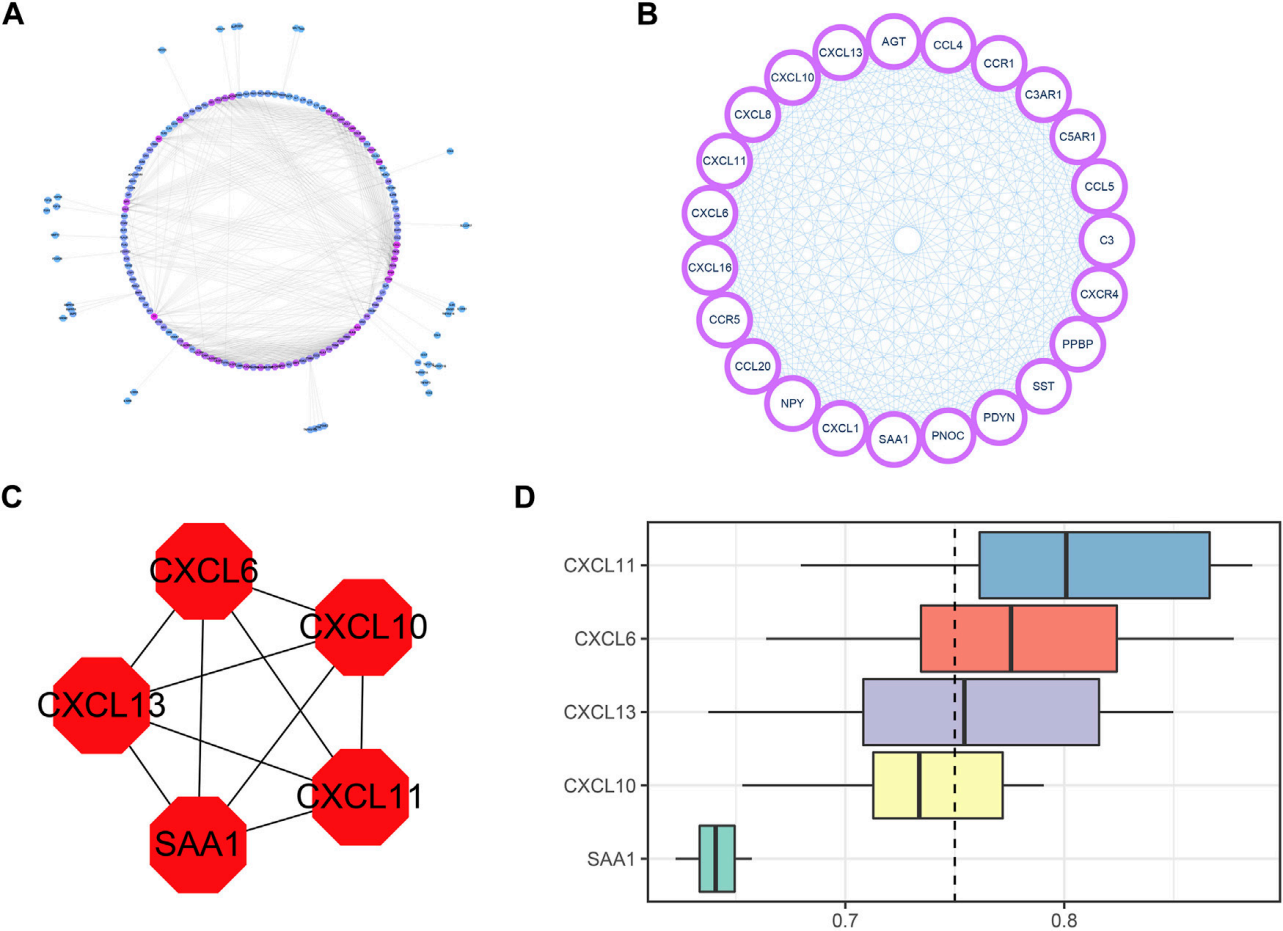
PPI network construction and hub gene mining. **(A)** PPI network diagram with DEIRGs interaction fraction greater than or equal to 0.9, including 156 nodes and 810 edges. The more purple the color is, the greater the node degree is, and the bluer the color is, the smaller the node degree is. **(B)** The hub node mined by the MCODE plugin has a total of 23 nodes and 253 edges. **(C)** hub nodes excavated by cytoHubba plugin on the basis of MCODE. There are altogether 5 nodes and 10 edges. **(D)** GO enrichment cloud and rain map of 5 hub genes.

The network was constructed by the interaction of these DEIRGs with nodes with scores greater than or equal to 0.9 in the STRING database. Five hub genes, including CXCL6, CXCL10, CXCL11, CXCL13, and SAA1, were screened out by MCODE and cytoHubba plugins. PPI network interaction and hub gene mining are shown in Figure 5.

We ranked the top 5 genes based on the average functional similarity (Figure 5D), which showed that CXCL11 was the most important gene.

### Identification and Validation of Diagnostic Markers

**FIGURE 6:**
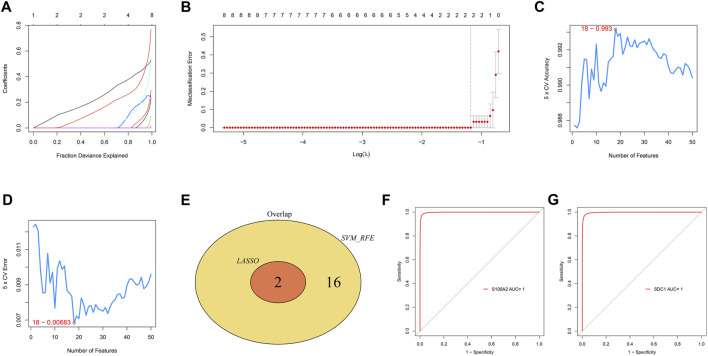
LASSO and Random Forest algorithms screening for diagnostic markers **(A,B)**. LASSO regression model accuracy curve based on Random Forest **(C)** and error curve based on Random Forest **(D)**. The Venn diagram shows the intersection of diagnostic markers obtained by the two algorithms **(E)**. The ROC curve of the diagnostic efficacy verification after fitting S100A2 **(F)** and SDC1 **(G)** in GSE94349.

In the GSE60815 dataset, the LASSO regression (Figures 6A,B) and Random Forest (Figures 6C,D) algorithms were used to screen diagnostic markers. Two diagnostic markers were selected by the LASSO method, and 18 were selected by the Random Forest method. The two diagnostic markers, namely S100A2 and SDC1, were selected as the final results (Figure 6E), and the S100A2 and SDC1 genes were analyzed by ROC in the external dataset GSE94349 (Figures 6F,G). The results showed that the Area Under Curve (AUC) values of S100A2 and SDC1 genes were both 1, so we selected S100A2 and SDC1 genes as the final diagnostic markers.

### Analysis of Immune Cell Infiltration and Its Correlation With Hub Genes Diagnostic Markers

**FIGURE 7:**
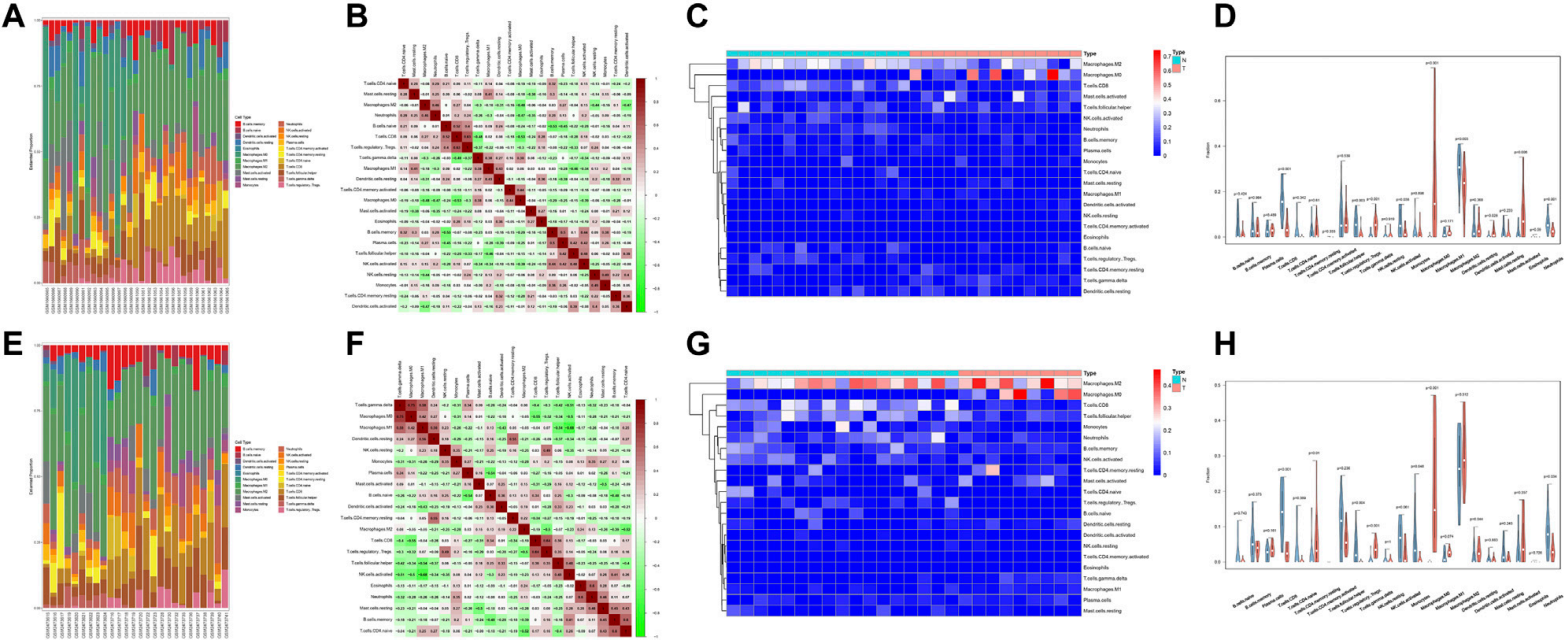
Evaluation and visualization of immune cell infiltration in GSE60815 datasets **(A–D)** and GSE94349 datasets **(E–H)**. Bar chart of immune infiltrating cells between ACP and control samples **(A,E)**. Correlation heat map of 22 types of immune cells. The size of the colored squares represents the strength of the correlation; blue represents a positive correlation; red represents a negative correlation. The darker the color, the stronger the correlation **(B,F)**. A heat map of immune cells between ACP and control samples **(C,G)**. Violin diagram of the proportion of 22 types of immune cells, and comparison of immune cells between the two ACP and control samples **(D,H)**.

The results of the heat map and bar chart of immune cell infiltration showed significant differences in immune infiltrating cells between ACP and control samples (Figures 7A,C,E,G). Violin diagram on immune cells infiltrating differences (Figures 7D,H) showed that, in ACP samples, M2 macrophages, activated NK cells, and gamma delta T cells infiltration were relatively more. In contrast, there were relatively few CD8+ T cells, regulatory T cells, Neutrophils, and activated mast cells infiltration. The heat map of 22 infiltrating immune cells showed that resting CD4+T cells were negatively correlated with gamma delta T cells and monocytes while positively correlated with resting dendritic cells and M1 macrophages (Figures 7B,F).

**FIGURE 8:**
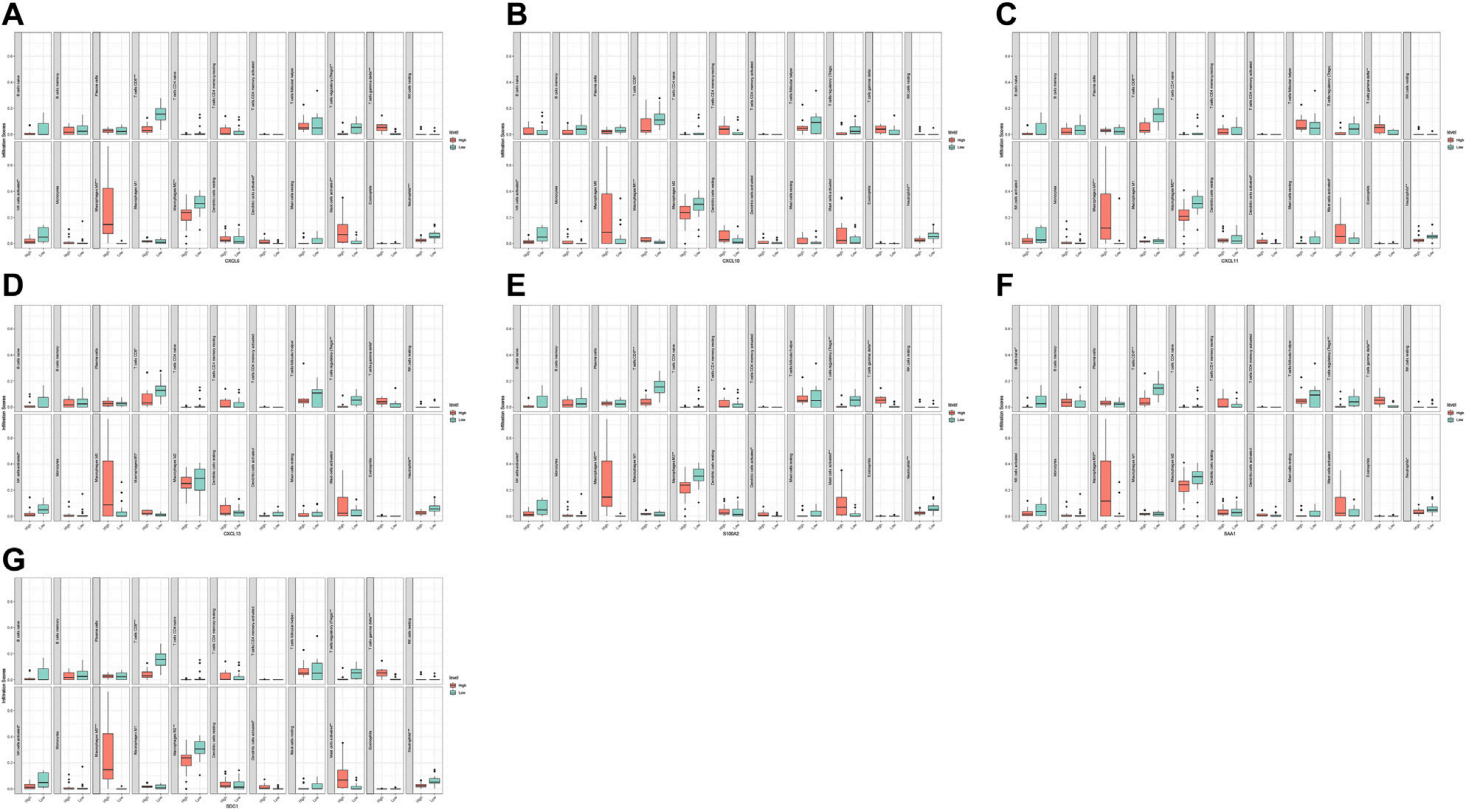
Correlation analysis of immune infiltrating cells with hub gene and diagnostic markers in GSE60815 dataset. CXCL6 **(A)**, CXCL10 **(B)**, CXCL11 **(C)**, CXCL13 **(D)**, S100A2 **(E)**, SAA1 **(F)**, and SDC1 **(G)**.

**FIGURE 9:**
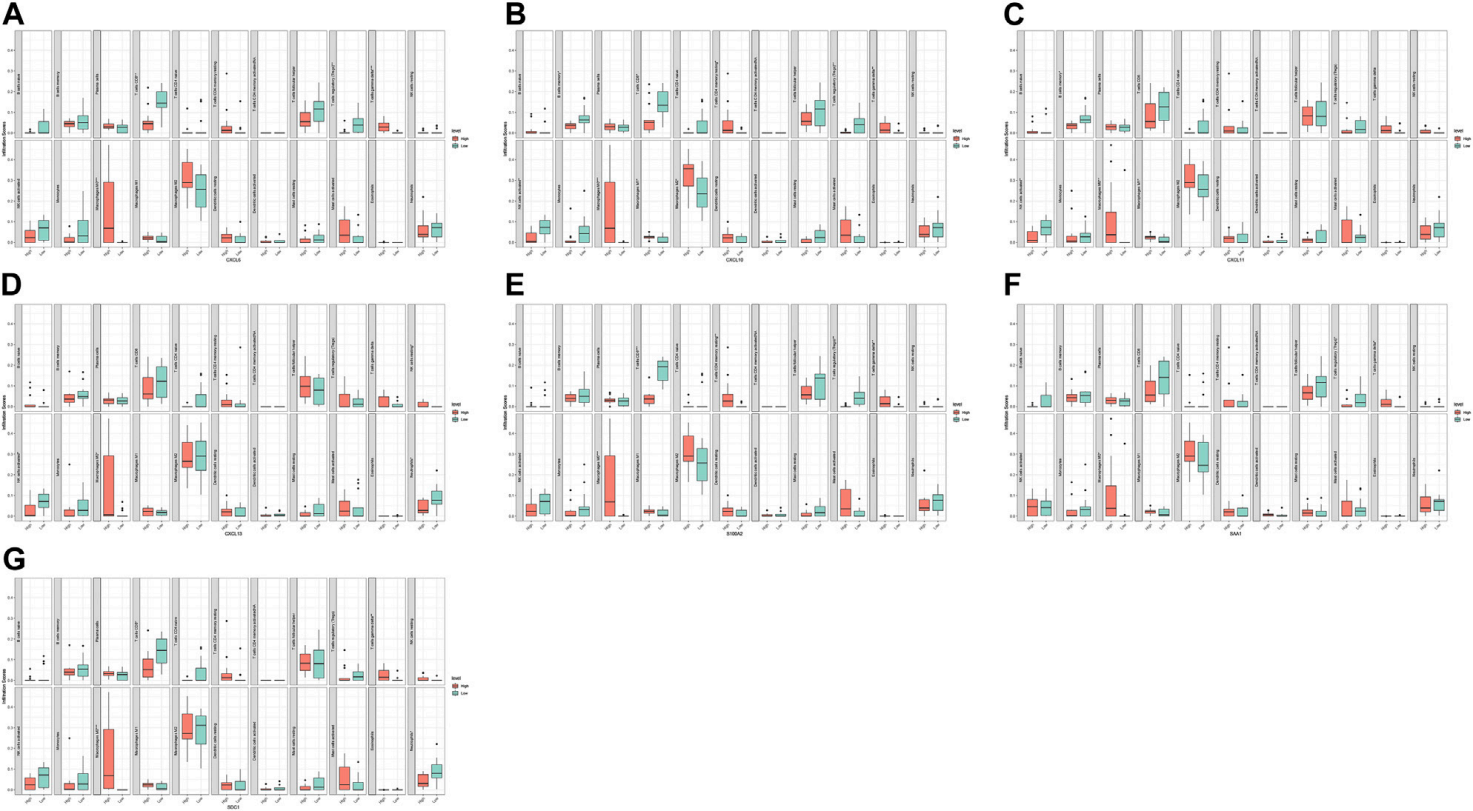
Correlation analysis of immune infiltrating cells with hub gene and diagnostic markers in GSE94349 dataset. CXCL6 **(A)**, CXCL10 **(B)**, CXCL11 **(C)**, CXCL13 **(D)**, S100A2 **(E)**, SAA1 **(F)**, and SDC1 **(G)**.

We analyzed and integrated the correlation between the five hub genes and the two diagnostic markers and immune infiltrating cells in two datasets, respectively. The results showed significant differences in various immune cells between the high and low groups with differential hub genes and diagnostic markers (Figures 8, 9). CXCL6 was positively correlated with gamma delta T cells and M0 macrophages but negatively correlated with CD8+ T cells and regulatory T cells; CXCL10 was positively correlated with M1 macrophages but negatively correlated with CD8+ T cells and activated NK cells; CXCL11 was positively correlated with M0 macrophages; CXCL13 was negatively correlated with activated NK cells and Neutrophils; SAA1 was positively correlated with gamma delta T cells and M0 macrophages but negatively correlated with regulatory T cells; S100A2 was positively correlated with gamma delta T cells and M0 macrophages but negatively correlated with CD8+ T cells and regulatory T cells; SDC1 was positively correlated with gamma delta T cells and M0 macrophages but negatively correlated with CD8+ T cells and Neutrophils.

### The Drug Sensitivity Analysis of Hub Genes and Diagnostic Marker

**FIGURE 10:**
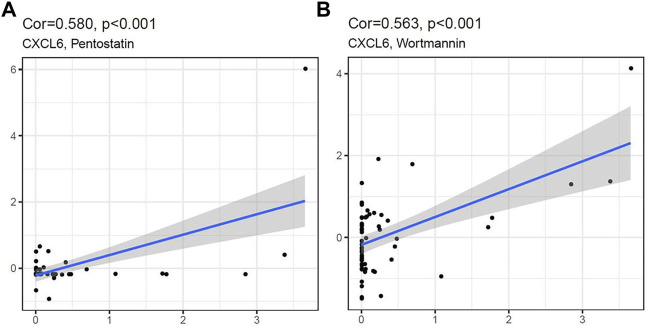
CXCL6 expression was positively correlated with Pentostatin **(A)** and Wortmannin **(B)** sensitivity.

We analyzed the relationship between the five hub genes and the two diagnostic markers and drug sensitivity. It showed that CXCL6 was significantly positively correlated with Pentostatin and Wortmannin, indicating that with the increase of CXCL6 expression (Figure 10), the sensitivity of the human body to Pentostatin and Wortmannin increased.

## Discussion

Although CP is a benign tumor, it is associated with a high mortality rate. According to the clinical research of Khafaga Y et al., the mortality rate of CP patients treated with surgery was as high as 16% (Khafaga et al., 1998). Harvey Cushing (Elliott et al., 2011) described CP as “one of the most baffling problems to the neurosurgeon.” CP includes two pathological types: ACP and PCP. The incidence of ACP is significantly higher than that of PCP, and the prognosis of ACP is worse than PCP (Adamson et al., 1990). Conventional surgery and radiation therapy for ACP are associated with complex complications and poor prognosis. This study aims to explore the biological mechanism of ACP and search for meaningful molecular markers, which may help find novel diagnosis methods and safe treatments for ACP.

In this study, we performed an integrative analysis of two expression profile datasets (GSE68015 and GSE94349), including 24 ACP samples and 33 normal samples. 271 DEIRGs in total were identified from these two expression profile datasets. Protein-protein interaction (PPI) network interaction and machine learning algorithm were used to screen out five hub genes and two diagnostic markers. CIBERSORT tools have also facilitated the analysis of immune cell infiltration patterns of ACP, and Gamma delta T cells and CD8+ T cells were found to be closely related to these molecular markers. CXCL6 expression was found positively correlated with the sensitivity of Pentostatin and Wortmannin in the Cellminer database. We sought to explore the potential molecular therapeutic targets and related molecular mechanisms of ACP and find a more optimal therapeutic approach.

A total of 271 DEIRGs were screened out in this study. To investigate the biological processes of the DEIRGs involved in ACP, we conducted enrichment analysis of GO, KEGG, and GSEA, respectively. GO enrichment analysis showed that leukocyte migration and cell chemotaxis signaling pathways were significantly associated with the occurrence and development of ACP. KEGG enrichment analysis showed that cytokine-cytokine receptor interaction and TNF signaling pathway were the significantly enriched pathways. Cytokines include chemokines, tumor necrosis factors (TNF), and interleukins. Chemokines may induce the directed migration of white blood cells, which is involved in inflammation and immune response in tumors (Balkwill, 2004). Gong et al. confirmed that the high expression of various cell chemokines in ACP was associated with poor recurrence-free survival (Gong et al., 2014). TNF-α plays an important role in tumor promotion and progression (Balkwill, 2006). Simone et al. suggested that TNF-α and IL-6 cells and other cytokines synergistically activate STAT3 and NF-κB pathways to promote colorectal cancer cell proliferation (De Simone et al., 2015). Mori et al. confirmed the expression of TNF-α in the cystic fluid of ACP tumors (Mori et al., 2004), which is consistent with our data mining results. Therefore, we believe that cytokines play an essential role in the immune response, inflammation, tumor cell proliferation, and apoptosis in ACP.

We screened out five hub genes, namely CXCL6, CXCL10, CXCL11, CXCL13, and SAA1, by constructing the PPI network. The first four of the five hub genes belong to chemokines, which, as key signaling molecules in the tumor microenvironment, play an important role in the interaction between tumor cells and tumor stromal cells (Balkwill, 2004). CXCL11 and CXCL10 are selective ligands for CXCR3, usually expressed at low levels under homeostasis but up-regulated under cytokine stimulation. CXCL11 and CXCL10 are secreted mainly by monocytes, endothelial cells, fibroblasts, and cancer cells in response to IFN-γ and are synergistically enhanced by IFN-α. Some literature suggests that CXCL11 and CXCL10 can promote the proliferation and metastasis of tumor cells (Cambien et al., 2009; Puchert et al., 2020). It has been reported that tumors with high expression of CXCL10 and CXCR3 simultaneously have stronger invasion and metastasis (Wightman et al., 2015). CXCL10, CXCL11/CXCR3 axis can be used as the target of tumor immunotherapy (Tokunaga et al., 2018). CXCL6, as another chemokine family member, also plays an important role in tumor proliferation and metastasis in various cancer, including non-small cell lung cancer (Li et al., 2018) and colon cancer (Ma et al., 2017). Zheng et al. reported that CXCL6 up-regulates PD-L1 expression by activating the STAT3 pathway, promoting the growth and metastasis of esophageal squamous cell carcinoma cells *in vivo* and *in vitro* (Zheng et al., 2021). CXCL13 and its chemokine receptor 5 (CXCR5) are among the key chemotactic factors which play crucial roles in deriving cancer cell biology (Hussain et al., 2019). In addition, the SAA1 gene encodes high-density lipoprotein-associated with inflammation, and high expression of SAA1 in pancreatic cancer predicts poor prognosis [39]. Zhang et al. pointed out that inhibiting the SAA1 gene could down-regulate the AKT signaling pathway and promote glioblastoma cell apoptosis, and SAA1 could be used as a potential therapeutic target and prognostic index of glioblastoma (Tokunaga et al., 2018).

LASSO Logistic and Random Forest Regressions were used to screen molecular diagnostic markers in the GSE68015 dataset, and the GSE94349 dataset was used to verify accuracy. Finally, two molecular diagnostic markers, namely S100A2 and SDC1, were selected. S100A2, a member of the S100 family, is a calcium-binding protein. Studies have confirmed that S100A2 participates in processes such as cell proliferation and migration in some tumors (Bulk et al., 2009; Wolf et al., 2011) A previous study showed that high expression of S100A2 in patients with non-small cell lung cancer has a worse prognosis (Wang et al., 2005). Another study indicated that the high expression of S100A2 is an independent prognostic marker for the poor prognosis of pancreatic cancer (Dreyer et al., 2020). Huang et al. suggested that down-regulating S100A2 was able to inhibit the Wnt/β-Catenin signaling pathway, thus reliving the epithelial-mesenchymal transition (EMT) in pulmonary fibrosis (Huang et al., 2021). The accumulation of β-catenin in the nucleus, which further leads to activation of the Wnt pathway, is the main pathogenic mechanism of ACP (Larkin and Ansorge, 2013). We speculate that S100A2 plays an important role in the pathogenesis of ACP. SDC1 is a type I transmembrane glycoprotein mainly expressed in epithelial cells, and disorder of SDC1 expression leads to cancer development by promoting cell proliferation, metastasis, invasion, and angiogenesis. The expression of SDC1 varies in different types of tumors. SDC1 is low expressed in gastric cancer and colorectal cancer, whereas SDC1 is highly expressed in plasmacytoid urothelial carcinoma, liver cancer, and glioma (Liao et al., 2020). Chen et al. reported that SDC1 overexpression significantly increased glioma cells migration, while SDC1 knockdown had the opposite effects (Chen et al., 2017). Another study confirmed that SDC1 knockdown inhibits glioma cell proliferation and invasion by deregulating a c-src/FAK-associated signaling pathway (Shi S. et al., 2017). Therefore, we believe that SDC1 may play a key role in the pathogenetic process of ACP and may serve as a potential therapeutic target in the treatment of ACP.

In this study, the CIBERSORT is used for the first time to analyze the immune cell infiltration in ACP and normal brain tissue to explore the role of tumor-infiltrating immune cells in ACP. We found that increased infiltration of M2 macrophages, activated NK cells, and a decreased infiltration of regulatory T cells, Neutrophils may be related to the occurrence and development of ACP. Macrophages can be polarised towards either M1macrophages or M2 macrophages, and M2 macrophages play an important role in the proliferation of CNS tumors. Komohara et al. reported that M2 macrophages promote the proliferation of glioma cells through M-CSFR/Stat3 (Komohara et al., 2012). In another study, we found that the expression of M2 macrophages in the recurrent CP samples was higher than that in the primary and recurrent CP samples of the same patient, and the more expression of M2 macrophages in the recurrent CP samples, the shorter the recurrence time of the tumor (Lin et al., 2019). We conclude that M2 macrophages play an important role in ACP tumor proliferation. In this study, the proportion of neutrophils in ACP samples was low and significantly lower than that in the normal control group, which was consistent with the results of Coy S et al. (Coy et al., 2018).

By analyzing the correlation between the hub genes and diagnostic markers and immune cells, it was found that CXCL6, S100A2, SDC1, SAA1 were significantly positively correlated with gamma delta T cells. CXCL6, S100A2, SDC1, CXCL10 were significantly negatively correlated with CD8+ T cells. In recent years, studies have confirmed that gamma delta T cells play an important role in the occurrence and progression of tumors (Fleming et al., 2017), while CD8+ T cells are associated with the prognosis of patients and the outcome of immunotherapy. Daley et al. confirmed that tumor-infiltrating gamma delta T cells promote tumor progression through the PD-1/PD-L1 pathway restraining αβ T cell activation (Daley et al., 2016). Gamma delta T17 cell is the main manufacturer of IL-17. Coffelt et al. confirmed in a breast cancer model that IL-17 might induce tumor immunosuppression and promote tumor neovascularization, among which, Il-17 inhibits the activity of CD8+T cells by regulating the tumor-infiltrating neutrophils (Coffelt et al., 2015). We hypothesized that CXCL6, S100A2, and SDC1 lead to tumor progression and immune escape by raising gamma delta T cells and reducing CD8+T cells, but this hypothesis needs further verification.

Finally, we used CellMiner to analyze the drug sensitivity of hub gene and diagnostic markers, and we found that with the increase of CXCL6 expression, the drug sensitivity to Pentostatin and Wortmannin increased. We believe that in ACP with high CXCL6 expression, Pentostatin and Wortmannin can be used as potential molecular targeted drugs for treatment, but the mechanism of action between drugs and molecules needs to be confirmed by further studies.

With the advent of the era of molecular therapy, the treatment of ACP requires more correlation studies on the precise molecular targeted therapy, especially for complicated and rapidly-recurring ACPs and those not suitable for surgical treatment. Grob et al. found that tocilizumab and bevacizumab could reduce tumor volume in cystic ACP with high IL-6 expression (Grob et al., 2019). Apps et al. reported that inhibition of MAPK/ERK signaling pathway by MEK inhibitors could reduce the proliferation of ACP tumor cells or increase tumor cell apoptosis (Apps et al., 2018). These studies, including this one, provide significant clues and evidence for the feasibility of molecularly targeted therapy for ACP.

There are some limitations in the current study. First, ACP includes the solid component, cystic wall, cystic fluid, calcification, and other pathological tissue components. The expression profile data were not precisely selected from specific tumor components, and the gene expression may differ with different tumor tissue components. Second, this study should apply basic experiments to verify the expression of the excavated hub gene and diagnostic marker gene. For example, the role of hub genes and diagnostic markers should be fully elucidated through real-time PCR, immunohistochemistry, immunofluorescence. Third, due to the lack of clinical characteristics data of ACP patients in the original data mining, it is not possible to carry out the correlation study of hub gene, diagnostic markers, and immune infiltrating cells with clinical characteristics of ACP, such as imaging characteristics, recurrence, and survival rates.

Through integrative analysis of biomarkers and mechanisms in ACP expression profile datasets in this study, we believe that the results may provide new insights into the diagnosis and treatment of ACP. Although some results of the previous studies are consistent with the results of our analysis, further experimental validation on the reliability of the results is needed.

## Conclusion

In summary, we found five hub genes, namely CXCL6, CXCL10, CXCL11, CXCL13, and SAA1, and two diagnostic markers, namely S100A2 and SDC1, play a crucial role in the pathogenesis of ACP. We also found that M2 macrophages, activated NK cells, gamma delta T cells, CD8+ T cells, regulatory T cells, Neutrophils, and activated mast cells were involved in the occurrence and progression of ACP. Among them, gamma delta T cells were significantly positively correlated with CXCL6, S100A2, SDC1, and SAA1, while CD8+ T cells were significantly negatively correlated with CXCL6, S100A2, SDC1, and CXCL10. Moreover, ACP with high CXCL6 expression showed remarkable drug sensitivity to Pentostatin and Wortmannin; thus, we believe that Pentostatin and Wortmannin can be used as potential molecular targeted drugs to treat ACP with high CXCL6 expression, but the specific drug mechanism needs further verification. The hub genes, diagnostic markers, and immune cells identified in this study may serve as targets for molecular therapy or immunotherapy and provide guidance for developing a novel and safe treatment for ACP.

## Data Availability

The datasets presented in this study can be found in online repositories. The names of the repository/repositories and accession number(s) can be found in the article/Supplementary Material.
